# Data on factors characterizing the eLearning experience of secondary school teachers and university undergraduate students in Jordan

**DOI:** 10.1016/j.dib.2020.106402

**Published:** 2020-10-10

**Authors:** Ahmad Hamza Obidat, Mahmoud Alquraan, Malak Hamza Obeidat

**Affiliations:** a Al-Hussein Bin Talal University, Jordan; b Yarmouk University, Jordan

**Keywords:** eLearning, Educational technology, Technology acceptance model, eLearning satisfaction model, University undergraduate students, Secondary school teachers, COVID-19, Jordan

## Abstract

Two datasets were obtained to develop an understanding of factors characterizing the eLearning experience of secondary school teachers and university undergraduate students in Jordan. Both datasets were collected via electronic questionnaires. The secondary school teachers’ dataset was collected toward the end of the second semester of the 2019/2020 academic year, and the university undergraduate students’ dataset was collected during the summer semester of the same year. Six hundred and sixty-six participants responded to the secondary school teachers’ questionnaire, and one thousand participants responded to the university undergraduate students’ questionnaire. A structural equation modelling approach was utilized to analyze the data. Future research might extend the two data models by collecting data about other factors and examine their impact on the eLearning experience of secondary school teachers and university undergraduate students in Jordan and other countries. Moreover, the datasets can be compared with other data from different developing and developed countries.

## Specifications Table

**Table:** 

Subject	Education
Specific subject area	Educational technology
Type of data	Table
How data were acquired	Surveys: two electronic questionnaires were put out seeking responses from secondary school teachers and university undergraduate students in Jordan about their eLearning experience
Data format	Raw Analyzed
Parameters for data collection	Two datasets were obtained to get an understanding of factors characterizing the eLearning experience of secondary school teachers and university undergraduate students in Jordan. The secondary school teachers’ dataset was collected toward the end of the second semester of the 2019/2020 academic year, and the university undergraduate students’ dataset was collected during the summer semester of the same year. For secondary school teachers, a questionnaire was distributed targeting teachers who teach from eighth grade to twelfth grade, those teachers used Microsoft Teams to deliver instruction as mandated by the Jordanian Ministry of Education during COVID-19 lockdown in Jordan. For university undergraduate students, a questionnaire was put out seeking responses from university undergraduate students during that summer semester, where the Jordanian Ministry of Higher Education mandated that all courses for undergraduate students to be taught online to prevent the diffusion of COVID-19 disease
Description of data collection	Hosted on Google forms, both datasets were collected via two different electronic questionnaires. The invitation letters to participate, in the questionnaires, were distributed via social networks of secondary school teachers and public and private university undergraduate students in Jordan. Six hundred and sixty-six participants responded to the school teachers’ questionnaire, and one thousand responded to the university undergraduate students’ questionnaire
Data source location	Secondary schools and public and private universities in Jordan
Data accessibility	Both datasets are with the article

## Value of the Data

• The datasets are important because they capture data about factors characterizing the eLearning experience of secondary school teachers and university undergraduate students in Jordan, which is useful in formulating an eLearning strategy in Jordan and other countries.
• Online course developers, university instructors, school teachers, administrators, educational policy makers, and learning management system vendors can utilize the datasets in support of their decision-making processes.
• Future research might extend the two data models by collecting data about other factors and examine their impact on the eLearning experience of secondary school teachers and university undergraduate students in Jordan and other countries. Moreover, the datasets can be compared with other data from different developing and developed countries.

## 1 Data Description

The data for secondary school teachers, (Appendix A), was collected toward the end of the second semester of the 2019/2020 schools academic year in Jordan. Such that, a questionnaire (Appendix B) was distributed targeting secondary school teachers – teachers who teach from eighth grade to twelfth grade. Those teachers used Microsoft Teams to deliver instruction as mandated by the Jordanian Ministry of Education, during COVID-19 lockdown in Jordan. The questionnaire was hosted on a Google form. The invitation to participate in the electronic questionnaire was distributed via social networks of secondary school teachers in Jordan. Six hundred and sixty-six teachers filled-in the questionnaire. Table 1 presents the demographic characteristics of secondary school teacher participants.

**Table 1.** Demographic characteristics of secondary school teacher participants.

	Frequency	Percentage
**Gender**		
Male	236	35.4%
Female	430	64.6%
Total	666	100%
**Age**		
20–29	70	10.5%
30–39	196	29.4%
40–49	322	48.3%
50–59	76	11.5%
60–70	2	0.3%
Total	666	100%
**Education**		
Community college	34	5%
Bachelor	403	61%
Postgraduate diploma	121	18%
Master	77	11.5%
Doctorate	31	4.5%
Total	666	100%

The data for university undergraduate students, (Appendix C), was collected during the summer semester of 2019/2020 academic year in Jordan. Hosted on a Google form, a questionnaire (Appendix D) was put out seeking responses from public and private university undergraduate students in Jordan about their eLearning experience. The invitation letter to participate in the electronic questionnaire was sent via social networks of public and private university undergraduate students in Jordan. One thousand responses to the questionnaire were obtained. Table 2 presents the demographic characteristics of university undergraduate student participants.

**Table 2.** Demographic characteristics of university undergraduate student participants.

	Frequency	Percentage
**Gender**		
Male	490	49%
Female	510	51%
Total	1000	100%
**Age**		
18–20	391	39.1%
21–23	456	45.6%
24–26	96	9.6%
27–29	57	5.7%
Total	1000	100%
**Academic level**		
First year	230	23%
Second year	218	21.8%
Third year	253	25.3%
Fourth year	234	23.4%
Fifth year	47	4.7%
Sixth year	18	1.8%
Total	1000	100%

For both datasets, all constructs were operationalized using validated items from prior research. Table 3 presents the constructs for both datasets, their associated number of items, and the resources they were obtained from.

**Table 3.** Constructs for the first and second datasets.

Construct	Number of items	Resource	Teachers’ dataset	Students’ dataset
Perceived ease of use	4	[1]	✓	✓
Perceived usefulness	4	[1]	✓	✓
Intention to continuous use	2	[1]	✓	✓
Attitude toward use	4	[2]	✓	
Subjective norms	2	[2]	✓	✓
Facilitating conditions	5	[3]	✓	
Computer self-efficacy	10	[4]	✓	
Computer anxiety	4	[5]	✓	
User satisfaction	4	[6]	✓	✓
Complexity	4	[7]	✓	
Outcome expectations	7	[8]	✓	
Compatibility	3	[9]	✓	
Technical support	3	[10]	✓	
Learning community	3	[11]		✓
Learning content	4	[11]		✓
Learning personalization	4	[11]		✓

To establish convergent validity for each measurement model, four criteria were assessed: (1) standardized Factor Loading (FL) for each item which must be greater than 0.5, (2) composite reliability (CR) for each construct which must be greater than 0.7, (3) Cronbach's alpha for each construct which must be greater than 0.7, and (4) the average variance extracted (AVE) for each construct which must be above 0.5 [12]. With respect to secondary school teachers’ measurement model, the absolute value of FL for the fifth item measuring the facilitating conditions construct (FC5), which is reverse coded, was below the specified threshold. As such, the item was removed from the model. By examining Table 4, it can be concluded that convergent validity of secondary school teachers’ measurement model has been established after removing FC5. Also, by examining Table 5, it can be concluded that convergent validity of university undergraduate students’ measurement model has been established as well.

**Table 4.** Secondary school teachers’ measurement model assessment.

Construct	Item	Cronbach's *α*	CR	AVE	FL				

Perceived ease of use	PE1	0.89	0.89	0.68	0.81				
	PE2				0.80				
	PE3				0.81				
	PE4				0.88				
Perceived usefulness	PU1	0.94	0.94	0.80	0.86				
	PU2				0.91				
	PU3				0.91				
	PU4				0.89				
User satisfaction	US1	0.95	0.93	0.82	0.89				
	US2				0.95				
	US3				0.89				
	US4				0.88				
Subjective norms	SN1	0.87	0.87	0.77	0.85				
	SN2				0.90				
Attitude toward use	AT1	0.92	0.86	0.70	0.79				
	AT2				0.79				
	AT3				0.87				
	AT4				0.86				
Intention to continuous use	IC1	0.93	0.93	0.87	0.93				
	IC2				0.93				
Computer self-efficacy	CS1	0.94	0.93	0.62	0.60				
	CS2				0.56				
	CS3				0.77				
	CS4				0.82				
	CS5				0.86				
	CS6				0.88				
	CS7				0.84				
	CS8				0.81				
	CS9				0.85				
	CS10				0.82				
Outcome expectations	OE1	0.91	0.87	0.57	0.85				
	OE2				0.70				
	OE3				0.91				
	OE4				0.81				
	OE5				0.75				
	OE6				0.65				
	OE7				0.56				
Computer anxiety	CA1	0.88	0.88	0.65	0.75				
	CA2				0.72				
	CA3				0.87				
	CA4				0.86				
Complexity	CX1	0.84	0.85	0.58	0.72				
	CX2				0.73				
	CX3				0.78				
	CX4				0.82				
Compatibility	CT1	0.91	0.91	0.78	0.85				
	CT2				0.91				
	CT3				0.89				

Construct	Item	Cronbach's α	CR	AVE	FL				
Technical support	TS1	0.80	0.82	0.61	0.57				
	TS2				0.84				
	TS3				0.88				

		Before item removal				After item removal			
		Cronbach's α	CR	AVE	FL	Cronbach's α	CR	AVE	FL
Facilitating conditions	FC1	0.75	0.82	0.55	0.83	0.87	0.88	0.65	0.83
	FC2				0.75				0.75
	FC3				0.86				0.86
	FC4				0.77				0.77
	FC5				**−0.09**				a

a Item removed.

**Table 5.** University undergraduate students’ measurement model assessment.

Constructs	Items	Cronbach's α	CR	AVE	FL
Perceived ease of use	PE1	0.88	0.88	0.66	0.80
	PE2				0.82
	PE3				0.79
	PE4				0.82
Perceived usefulness	PU1	0.93	0.93	0.77	0.87
	PU2				0.87
	PU3				0.89
	PU4				0.87
Subjective norms	SN1	0.85	0.85	0.74	0.83
	SN2				0.88
Intention to continuous use	IC1	0.91	0.91	0.83	0.91
	IC2				0.91
Learning community	LM1	0.87	0.88	0.65	0.74
	LM2				0.71
	LM3				0.85
	LM4				0.90
Learning content	LN1	0.87	0.87	0.70	0.78
	LN2				0.89
	LN3				0.83
Learning personalization	LP1	0.89	0.89	0.67	0.81
	LP2				0.80
	LP3				0.84
	LP4				0.82
User satisfaction	US1	0.95	0.95	0.83	0.90
	US2				0.91
	US3				0.92
	US4				0.91

The discriminant validity for each model was assessed by the heterotrait–monotrait (HTMT) ratio of correlations as it is the most rigor approach for achieving that goal [13]. The criterion in this approach is that the HTMT for each pair of constructs must be below 0.9 [13]. As presented in Tables 6 and 7, in both models, the HTMT for each pair of constructs is below 0.9. As such, the discriminant validity for each measurement model has been established.

**Table 6.** HTMT matrix for secondary school teachers’ measurement model.

Construct	PE	PU	US	SN	AT	IC	CS	OE	CA	CX	CT	TS	FC
PE	1.00												
PU	0.72	1.00											
US	0.68	0.70	1.00										
SN	0.54	0.71	0.61	1.00									
AT	0.65	0.70	0.73	0.60	1.00								
IC	0.65	0.74	0.77	0.61	0.82	1.00							
CS	0.58	0.68	0.63	0.64	0.65	0.71	1.00						
OE	0.56	0.81	0.78	0.69	0.71	0.79	0.69	1.00					
CA	0.32	0.12	0.18	0.05	0.24	0.18	0.10	0.11	1.00				
CX	0.49	0.34	0.40	0.25	0.38	0.37	0.17	0.31	0.60	1.00			
CT	0.60	0.75	0.80	0.68	0.70	0.78	0.63	0.88	0.12	0.39	1.00		
TS	0.57	0.61	0.71	0.55	0.57	0.61	0.56	0.69	0.16	0.29	0.69	1.00	
FC	0.82	0.66	0.77	0.58	0.64	0.70	0.69	0.63	0.32	0.44	0.68	0.74	1.00

Note. PE: Perceived ease of use, PU: Perceived usefulness, US: User satisfaction, SN: Subjective norms, AT: Attitude toward use, IC: Intention to continuous use, CS: Computer self-efficacy, OE: Outcome expectations, CA: Computer anxiety, CX: Complexity, CT: Compatibility, TS: Technical support, FC: Facilitating conditions.

**Table 7.** HTMT matrix for university undergraduate students’ model.

Constructs	PE	PU	SN	IC	LM	LN	LP	US
PE	1.00							
PU	0.74	1.00						
SN	0.69	0.74	1.00					
IC	0.72	0.85	0.70	1.00				
LM	0.74	0.71	0.65	0.71	1.00			
LN	0.71	0.68	0.60	0.64	0.85	1.00		
LP	0.76	0.80	0.69	0.77	0.83	0.84	1.00	
US	0.75	0.80	0.65	0.83	0.75	0.72	0.80	1.00

Note. PE: Perceived ease of use, PU: Perceived usefulness, SN: Subjective norms, IC: Intention to continuous use, LM: Learning community, LN: Learning content, LP: Learning personalization, US: User satisfaction.

To confirm the goodness-of-fit for each model, five criteria were used: (1) Comparative Fit Index (CFI) [14], (2) Tucker-Lewis Index (TLI) [15], (3) Relative Non-Centrality Index (RNI) [15], (4) Root Mean Square Error of Approximation (RMSEA) [16], and (5) Standardized Root Mean Square Residual (SRMR) [17]. Each model met the thresholds of all criteria as shown in Table 8.

**Table 8.** Fit indices of secondary school teachers and university undergraduate students’ structural models.

Fit indices	Teachers’ model	Students’ model	Criteria
CFI	0.928	0.959	>0.9
TLI	0.921	0.953	>0.9
RNI	0.928	0.959	>0.9
RMSEA	0.052	0.058	<.08
SRMR	0.074	0.037	<.08

Structural equation modeling approach, in R language version 4.0.1, was applied to analyze the data for each model. Appendix E and Appendix F have the R code for analyzing secondary school teachers and university undergraduate students’ datasets, respectively. Table 9 presents the analysis results of the university undergraduate students’ structural model, and Table 10 presents the analysis results of the secondary school teachers’ structural model.

**Table 9.** University undergraduate students’ structural model analysis results.

Path	Coefficient	*z*-value	*P*(>|*z*|)	Decision
Subjective norms→ Perceived usefulness	0.340	7.966	0.000	Significant
Learning content → Perceived usefulness	−0.046	−0.747	0.455	Insignificant
Learning community → Perceived usefulness	0.072	1.262	0.207	Insignificant
Learning personalization → Perceived usefulness	0.499	7.596	0.000	Significant
Perceived ease of use → Perceived usefulness	0.189	4.168	0.000	Significant
Subjective norms → Perceived ease of use	0.296	7.161	0.000	Significant
Learning community → Perceived ease of use	0.174	2.951	0.003	Significant
Learning content → Perceived ease of use	0.133	2.099	0.036	Significant
Learning personalization → Perceived ease of use	0.341	5.303	0.000	Significant
Subjective norms → Intention to continuous use	0.114	2.490	0.013	Significant
Perceived usefulness → Intention to continuous use	0.517	10.609	0.000	Significant
Perceived ease of use→ Intention to continuous use	0.072	1.588	0.112	Insignificant
User satisfaction → Intention to continuous use	0.365	8.982	0.000	Significant
Subjective norms→ User satisfaction	0.006	0.123	0.902	Insignificant
Perceived usefulness → User satisfaction	0.588	13.647	0.000	Significant
Perceived ease of use → User satisfaction	0.423	9.676	0.000	Significant

**Table 10.** Secondary school teachers’ structural model analysis results.

Path	Coefficient	*z*-value	*P*(>|*z*|)	Decision
Subjective norms → Perceived usefulness	0.367	8.943	0.000	Significant
Computer self-efficacy → Perceived usefulness	0.231	4.639	0.000	Significant
Technical support → Perceived usefulness	0.161	3.391	0.001	Significant
Complexity → Perceived usefulness	−0.019	−0.545	0.585	Insignificant
Perceived ease of use → Perceived usefulness	0.375	9.458	0.000	Significant
Subjective norms → Perceived ease of use	0.085	1.407	0.159	Insignificant
Computer self-efficacy → Perceived ease of use	0.344	5.302	0.000	Significant
Technical support → Perceived ease of use	0.599	4.441	0.000	Significant
Complexity → Perceived ease of use	−0.342	−7.360	0.000	Significant
Perceived usefulness → User satisfaction	−0.075	−1.057	0.291	Insignificant
Perceived ease of use → User satisfaction	0.100	1.674	0.094	Insignificant
Facilitating conditions → User satisfaction	0.361	6.218	0.000	Significant
Compatibility → User satisfaction	0.355	4.956	0.000	Significant
Outcome expectations → User satisfaction	0.341	3.967	0.000	Significant
Computer anxiety → User satisfaction	−0.003	−0.104	0.917	Insignificant
Subjective norms → User satisfaction	0.001	0.020	0.984	Insignificant
Perceived usefulness → Attitude toward use	−0.064	−0.616	0.538	Insignificant
Perceived ease of use → Attitude toward use	0.337	3.831	0.000	Significant
Facilitating conditions → Attitude toward use	−0.010	−0.122	0.903	Insignificant
Compatibility → Attitude toward use	0.079	0.773	0.439	Insignificant
Outcome expectations → Attitude toward use	0.746	5.860	0.000	Significant
Computer anxiety → Attitude toward use	−0.180	−4.399	0.000	Significant
Subjective norms → Attitude toward use	0.152	2.041	0.041	Significant
Perceived usefulness → Intention to continuous use	0.223	4.018	0.000	Significant
Perceived ease of use → Intention to continuous use	−0.032	−0.653	0.513	Insignificant
Attitude toward use → Intention to continuous use	0.477	11.589	0.000	Significant
User satisfaction → Intention to continuous use	0.274	6.703	0.000	Significant
Subjective norms → Intention to continuous use	0.009	0.178	0.859	Insignificant

## 2 Experimental Design, Materials and Methods

Two datasets were obtained to get an understanding of factors characterizing the eLearning experience of secondary school teachers and university undergraduate students in Jordan. The first dataset is concerned with secondary school teachers, and the second dataset is concerned with university undergraduate students. The first and second datasets were collected via two different questionnaires. Since the language of all prospected respondents is Arabic, both questionnaires were translated into their native Arabic language. Attitude toward use in the first dataset, and user satisfaction in both datasets were operationalized using a 5-point semantic differential scale from 1 to 5, with higher numbers reflecting more positive evaluations. The rest of the constructs, in both datasets, were operationalized using a 5-point Likert scale from 1 (“strongly disagree”) to 5 (“strongly agree”). Structural equation modeling approach, in R language version 4.0.1, was applied to analyze the data for each model.

## Ethics Statement

Ethical approvals for collecting both datasets were obtained from Al-Hussein Bin Talal University in Jordan. In the first page of both electronic questionnaires, the objectives of the research were presented, stating clearly that participation was voluntary, and the answers would be kept confidential. Informed consent of all participants was obtained before participating in either questionnaire.

## Declaration of Competing Interest

The authors declare that they have no known competing financial interests or personal relationships which have, or could be perceived to have, influenced the work reported in this article.

## Appendix Supplementary materials

Image, application 1

Image, application 2

Image, application 3

Image, application 4

Image, application 5

Image, application 6
