# Supplementary material for: Data on factors characterizing the eLearning experience of secondary school teachers and university undergraduate students in Jordan
Source: Data Brief. 2020 Oct 10;33:106402. doi: 10.1016/j.dib.2020.106402 (PMC7547838; doi:10.1016/j.dib.2020.106402)
Supplement: Supplementary file 2 [file mmc2.docx]

**Questionnaire for Secondary School Teachers**

What is your gender (Male/Female)?

How old are you?

What is the highest academic qualification you have? Please select one:

- Doctorate
- Master
- Postgraduate diploma
- Bachelor
- Community college

Perceived Ease of Use (PE)

1. Learning to operate Microsoft Teams would be easy for me
   1. Strongly disagree 2. Disagree 3. Neutral 4. Agree 5. Strongly agree
2. I would find it easy to get Microsoft Teams to do what I want it to do
   1. Strongly disagree 2. Disagree 3. Neutral 4. Agree 5. Strongly agree
3. It would be easy for me to become skillful at using Microsoft Teams

1. Strongly disagree 2. Disagree 3. Neutral 4. Agree 5. Strongly agree

1. I would find Microsoft Teams easy to use
   1. Strongly disagree 2. Disagree 3. Neutral 4. Agree 5. Strongly agree

Perceived Usefulness (PU)

1. Using Microsoft Teams would improve my teaching performance
   1. Strongly disagree 2. Disagree 3. Neutral 4. Agree 5. Strongly agree
2. Using Microsoft Teams in teaching would increase my productivity in teaching

1. Strongly disagree 2. Disagree 3. Neutral 4. Agree 5. Strongly agree

1. Using Microsoft Teams would enhance my effectiveness in teaching
   1. Strongly disagree 2. Disagree 3. Neutral 4. Agree 5. Strongly agree
2. I would find Microsoft Teams useful in teaching
   1. Strongly disagree 2. Disagree 3. Neutral 4. Agree 5. Strongly agree

Subjective Norms (SN)

1. People who influence my behavior would think that I should use Microsoft Teams for teaching online
   1. Strongly disagree 2. Disagree 3. Neutral 4. Agree 5. Strongly agree
2. People who are important to me would think that I should use Microsoft Teams for teaching online
   1. Strongly disagree 2. Disagree 3. Neutral 4. Agree 5. Strongly agree

Attitude Toward Use (AT)

1. Using Microsoft Teams for teaching is a (1. Bad….2….3….4….5. Good) idea
2. Using Microsoft Teams for teaching is a (1. Foolish….2….3….4….5. Wise) idea
3. I (1. Dislike….2….3….4….5. Like) the idea of using Microsoft Teams for teaching
4. Using Microsoft Teams would be: (1. Unpleasant….2….3….4….5. Pleasant) for teaching

Intention to Continuous Use (IC)

1. Assuming I had access to Microsoft Teams, I intend to continue using it
   1. Strongly disagree 2. Disagree 3. Neutral 4. Agree 5. Strongly agree
2. Given that I had access to Microsoft Teams, I predict that I would continue using it
   1. Strongly disagree 2. Disagree 3. Neutral 4. Agree 5. Strongly agree

Computer Self-Efficacy (CS)

I Could Complete the Job Using Microsoft Teams:

1. if there was no one around to tell me what to do as I go
   1. Strongly disagree 2. Disagree 3. Neutral 4. Agree 5. Strongly agree
2. if I had never used a package like it before
   1. Strongly disagree 2. Disagree 3. Neutral 4. Agree 5. Strongly agree
3. if I had only the software manuals for reference
   1. Strongly disagree 2. Disagree 3. Neutral 4. Agree 5. Strongly agree
4. if I had seen someone else using it before trying it myself
   1. Strongly disagree 2. Disagree 3. Neutral 4. Agree 5. Strongly agree
5. if I could call someone for help if I got stuck
   1. Strongly disagree 2. Disagree 3. Neutral 4. Agree 5. Strongly agree
6. if someone else had helped me get started
   1. Strongly disagree 2. Disagree 3. Neutral 4. Agree 5. Strongly agree
7. if I had a lot of time to complete the job for which the software was provided
   1. Strongly disagree 2. Disagree 3. Neutral 4. Agree 5. Strongly agree
8. if I had just the built-in help facility for assistance
   1. Strongly disagree 2. Disagree 3. Neutral 4. Agree 5. Strongly agree
9. if someone showed me how to do it first
   1. Strongly disagree 2. Disagree 3. Neutral 4. Agree 5. Strongly agree
10. if I had used similar packages before this one to do the same job
    1. Strongly disagree 2. Disagree 3. Neutral 4. Agree 5. Strongly agree

Facilitating Conditions (FC)

1. I have control over using Microsoft Teams
   1. Strongly disagree 2. Disagree 3. Neutral 4. Agree 5. Strongly agree
2. I have the resources necessary to use Microsoft Teams
   1. Strongly disagree 2. Disagree 3. Neutral 4. Agree 5. Strongly agree
3. I have the knowledge necessary to use Microsoft Teams
   1. Strongly disagree 2. Disagree 3. Neutral 4. Agree 5. Strongly agree
4. Given the resources, opportunities, and knowledge it takes to use Microsoft Teams, it would be easy for me to use Microsoft Teams
   1. Strongly disagree 2. Disagree 3. Neutral 4. Agree 5. Strongly agree
5. Microsoft Teams is **not** compatible with other systems I use (Reverse Coded)
   1. Strongly disagree 2. Disagree 3. Neutral 4. Agree 5. Strongly agree

Computer Anxiety (CA)

1. I feel apprehensive about using computers
   1. Strongly disagree 2. Disagree 3. Neutral 4. Agree 5. Strongly agree
2. It scares me to think that I could cause the computer to destroy a large amount of information by hitting the wrong key
   1. Strongly disagree 2. Disagree 3. Neutral 4. Agree 5. Strongly agree
3. I hesitate to use a computer for fear of making mistakes that I cannot correct
   1. Strongly disagree 2. Disagree 3. Neutral 4. Agree 5. Strongly agree
4. Computers are somewhat intimidating to me
   1. Strongly disagree 2. Disagree 3. Neutral 4. Agree 5. Strongly agree

Complexity (CX)

1. Using Microsoft Teams takes too much time from my normal duties
   1. Strongly disagree 2. Disagree 3. Neutral 4. Agree 5. Strongly agree
2. Working with Microsoft Teams is so complicated, it is difficult to understand what is going on
   1. Strongly disagree 2. Disagree 3. Neutral 4. Agree 5. Strongly agree
3. Using Microsoft Teams involves too much time doing mechanical operations (e.g., data input)
   1. Strongly disagree 2. Disagree 3. Neutral 4. Agree 5. Strongly agree
4. It takes too long to learn how to use Microsoft Teams to make it worth the effort
   1. Strongly disagree 2. Disagree 3. Neutral 4. Agree 5. Strongly agree

Outcome Expectations (OE)

If I use the Microsoft Teams...

1. I will increase my effectiveness on teaching
   1. Strongly disagree 2. Disagree 3. Neutral 4. Agree 5. Strongly agree
2. I will spend less time on routine teaching tasks
   1. Strongly disagree 2. Disagree 3. Neutral 4. Agree 5. Strongly agree
3. I will increase the quality of output of my teaching
   1. Strongly disagree 2. Disagree 3. Neutral 4. Agree 5. Strongly agree
4. I will increase the quantity of output for the same amount of effort
   1. Strongly disagree 2. Disagree 3. Neutral 4. Agree 5. Strongly agree
5. My coworkers will perceive me as competent
   1. Strongly disagree 2. Disagree 3. Neutral 4. Agree 5. Strongly agree
6. I will increase my chances of obtaining a promotion
   1. Strongly disagree 2. Disagree 3. Neutral 4. Agree 5. Strongly agree
7. I will increase my chances of getting a raise
   1. Strongly disagree 2. Disagree 3. Neutral 4. Agree 5. Strongly agree

Compatibility (CT)

1. Using Microsoft Teams is compatible with all aspects of teaching
   1. Strongly disagree 2. Disagree 3. Neutral 4. Agree 5. Strongly agree
2. I think that using Microsoft Teams fits well with the way I like to work
   1. Strongly disagree 2. Disagree 3. Neutral 4. Agree 5. Strongly agree
3. Using the Microsoft Teams fits into my work style
   1. Strongly disagree 2. Disagree 3. Neutral 4. Agree 5. Strongly agree

Technical Support (TS)

1. I know where to turn to when I need any assistance with Microsoft Teams
   1. Strongly disagree 2. Disagree 3. Neutral 4. Agree 5. Strongly agree
2. In my school we get good technical support for Microsoft Teams
   1. Strongly disagree 2. Disagree 3. Neutral 4. Agree 5. Strongly agree
3. We have extensive support to help with problems related to Microsoft Teams
   1. Strongly disagree 2. Disagree 3. Neutral 4. Agree 5. Strongly agree

User satisfaction (US)

How do you feel about your overall experience of Microsoft Teams use?

1. Very dissatisfied ….2….3….4….5. Very satisfied
2. Very displeased….2….3….4….5. Very pleased
3. Very frustrated….2….3….4….5. Very contented
4. Absolutely terrible….2….3….4….5. Absolutely Delighted
