# Supplementary material for: Data on factors characterizing the eLearning experience of secondary school teachers and university undergraduate students in Jordan
Source: Data Brief. 2020 Oct 10;33:106402. doi: 10.1016/j.dib.2020.106402 (PMC7547838; doi:10.1016/j.dib.2020.106402)
Supplement: Supplementary file 4 [file mmc4.docx]

**Questionnaire for University Undergraduate Students**

**What is your gender (Male/Female)?**

**How old are you? (Please select one)**

- 18-20 years
- 21-23 years
- 24-26 years
- 27-29 years

**What is your academic level? (Please select one)**

- First year
- Second year
- Third year
- Fourth year
- Fifth year
- Sixth year

**Perceived Ease of Use (PE)**

1. Learning to operate the e-learning system would be easy for me
   1. Strongly disagree 2. Disagree 3. Neutral 4. Agree 5. Strongly agree
2. I would find it easy to get the e-learning system to do what I want it to do
   1. Strongly disagree 2. Disagree 3. Neutral 4. Agree 5. Strongly agree
3. It would be easy for me to become skillful at using the e-learning system
   1. Strongly disagree 2. Disagree 3. Neutral 4. Agree 5. Strongly agree
4. I would find the e-learning system easy to use
   1. Strongly disagree 2. Disagree 3. Neutral 4. Agree 5. Strongly agree

**Perceived Usefulness (PU)**

1. Using the e-learning system would improve my learning
   1. Strongly disagree 2. Disagree 3. Neutral 4. Agree 5. Strongly agree
2. Using the e-learning system in learning would increase my productivity in learning
   1. Strongly disagree 2. Disagree 3. Neutral 4. Agree 5. Strongly agree
3. Using the e-learning system would enhance my effectiveness in learning
   1. Strongly disagree 2. Disagree 3. Neutral 4. Agree 5. Strongly agree
4. I would find the e-learning system useful in learning
   1. Strongly disagree 2. Disagree 3. Neutral 4. Agree 5. Strongly agree

**Subjective Norm (SN)**

1. People who influence my behavior would think that I should use the e-learning system for learning online
   1. Strongly disagree 2. Disagree 3. Neutral 4. Agree 5. Strongly agree
2. People who are important to me would think that I should use the e-learning system for learning online
   1. Strongly disagree 2. Disagree 3. Neutral 4. Agree 5. Strongly agree

**Intention to Continuous Use (IC)**

1. Assuming I had access to the e-learning system, I intend to continue using it
   1. Strongly disagree 2. Disagree 3. Neutral 4. Agree 5. Strongly agree
2. Given that I had access to the e-learning system, I predict that I would continue using it
   1. Strongly disagree 2. Disagree 3. Neutral 4. Agree 5. Strongly agree

**Learning Community (LM)**

1. The e-learning system makes it easy for me to discuss questions with other students
   1. Strongly disagree 2. Disagree 3. Neutral 4. Agree 5. Strongly agree
2. The e-learning system makes it easy for me to access the shared content from the learning community
   1. Strongly disagree 2. Disagree 3. Neutral 4. Agree 5. Strongly agree
3. The e-learning system makes it easy for me to discuss questions with my instructors
   1. Strongly disagree 2. Disagree 3. Neutral 4. Agree 5. Strongly agree
4. The e-learning system makes it easy for me to share what I learn with the learning community
   1. Strongly disagree 2. Disagree 3. Neutral 4. Agree 5. Strongly agree

**Learning Content (LN)**

1. The e-learning system provides up to-date content
   1. Strongly disagree 2. Disagree 3. Neutral 4. Agree 5. Strongly agree
2. The e-learning system provides content that exactly fits my needs
   1. Strongly disagree 2. Disagree 3. Neutral 4. Agree 5. Strongly agree
3. The e-learning system provides sufficient content
   1. Strongly disagree 2. Disagree 3. Neutral 4. Agree 5. Strongly agree

**Learning Personalization (LP)**

1. The e-learning system enables me to learn the content I need
   1. Strongly disagree 2. Disagree 3. Neutral 4. Agree 5. Strongly agree
2. The e-learning system enables me to choose what I want to learn
   1. Strongly disagree 2. Disagree 3. Neutral 4. Agree 5. Strongly agree
3. The e-learning system enables me to control my learning progress
   1. Strongly disagree 2. Disagree 3. Neutral 4. Agree 5. Strongly agree
4. The e-learning system records my learning progress and performance
   1. Strongly disagree 2. Disagree 3. Neutral 4. Agree 5. Strongly agree

**User satisfaction (US)**

How do you feel about your overall experience of the e-learning system use?

1. Very dissatisfied ….2….3….4….5. Very satisfied
2. Very displeased….2….3….4….5. Very pleased
3. Very frustrated….2….3….4….5. Very contented
4. Absolutely terrible….2….3….4….5. Absolutely Delighted
