# Supplementary material for: Data on factors characterizing the eLearning experience of secondary school teachers and university undergraduate students in Jordan
Source: Data Brief. 2020 Oct 10;33:106402. doi: 10.1016/j.dib.2020.106402 (PMC7547838; doi:10.1016/j.dib.2020.106402)
Supplement: Supplementary file 5 [file mmc5.docx]

library(xlsx)

library (lavaan)

library(semTools)

library(dplyr)

Dataset <- read.xlsx("Teachers.xlsx", sheetIndex = 1,header=TRUE)

Data=data.matrix(Dataset, rownames.force = NA)

count(Dataset,Educational_Level)

agedata <- Dataset %>% mutate(agegroup = case_when(

Dataset$Age >= 60 & Dataset$Age <= 70 ~ '60-70',

Dataset$Age >= 50 & Dataset$Age <= 59 ~ '50-59',

Dataset$Age >= 40 & Dataset$Age <= 49 ~ '40-49',

Dataset$Age >= 30 & Dataset$Age <= 39 ~ '30-39',

Dataset$Age >= 20 & Dataset$Age <= 29 ~ '20-29'))

count(agedata, agegroup)

TAM.model <- ' PE =~ PE1 + PE2 + PE3 + PE4

PU =~ PU1 + PU2 + PU3 + PU4

US=~US1+US2+US3+US4

SN =~ SN1 + SN2

AT =~ AT1 + AT2 + AT3+ AT4

IC =~ IC1 + IC2

CS =~CS1 +CS2 +CS3 +CS4 +CS5 +CS6 +CS7 +CS8 +CS9 +CS10

OE =~OE1 +OE2 +OE3 +OE4 +OE5 +OE6+OE7

CA =~ CA1 + CA2 + CA3+ CA4

CX =~ CX1 + CX2 +CX3+ CX4

CT=~CT1 + CT2+ CT3

TS=~TS1+TS2+ TS3

FC =~FC1 +FC2 +FC3 +FC4

PU ~ SN+CS+CX+TS

PE ~ SN+CS+CX+TS

PU ~ PE

US ~ PU+ PE

US~FC+CT+OE+CA

AT~FC+CT+OE+CA

AT~ PU+ PE

IC~ PU+ PE

IC~ AT

IC~ US

US ~ SN

AT~ SN

IC~ SN

AT1 ~~ AT2

AT3 ~~ AT4

CS1 ~~ CS2

US3 ~~ US4

OE6 ~~ OE7

PU ~~ OE

PE ~~ FC

PE ~~ TS

'

SEM= sem(TAM.model, data=Data)

reliability(SEM)

htmt(TAM.model, data=Dataset)

fitmeasures(SEM)

summary(SEM,standardized=T,fit=T,rsquare=T)
