# Supplementary material for: Data on factors characterizing the eLearning experience of secondary school teachers and university undergraduate students in Jordan
Source: Data Brief. 2020 Oct 10;33:106402. doi: 10.1016/j.dib.2020.106402 (PMC7547838; doi:10.1016/j.dib.2020.106402)
Supplement: Supplementary file 6 [file mmc6.docx]

library(dplyr)

library(semTools)

library(xlsx)

library (lavaan)

Dataset <- read.xlsx("University_Students.xlsx", sheetIndex = 1,header=TRUE)

Data=data.matrix(Dataset, rownames.force = NA)

count(Dataset, Gender)

count(Dataset, Age)

count(Dataset, Academic_Year)

TAM.model <- '

Perceived_ease_of_use=~ PE1 + PE2 + PE3 + PE4

Perceived_usefulness=~ PU1 +PU2+PU3+PU4

Subjective_norms=~SN1+SN2

Intention_to_continuous_use =~ IC1+IC2

Learning_community=~LM1+LM2+LM3+LM4

Learning_content =~LN1+LN2+LN3

Learning_personalization=~LP1+LP2+LP3+LP4

User_satisfaction=~US1+US2+US3+US4

Perceived_usefulness ~Learning_community+Learning_content+Learning_personalization+Subjective_norms

Perceived_ease_of_use~Learning_community+Learning_content+Learning_personalization+Subjective_norms

User_satisfaction ~ Perceived_usefulness+ Perceived_ease_of_use

Intention_to_continuous_use ~ Perceived_usefulness+ Perceived_ease_of_use

Intention_to_continuous_use ~ Subjective_norms

Intention_to_continuous_use ~ User_satisfaction

User_satisfaction~ Subjective_norms

Perceived_usefulness ~Perceived_ease_of_use

'

SEM= sem(TAM.model, data=Data)

reliability(SEM)

htmt(TAM.model, data=Dataset)

fitmeasures(SEM)

summary(SEM,standardized=T,fit=T,rsquare=T)
